# Comparative Studies of Selected Criteria Enabling Optimization of the Extraction of Polar Biologically Active Compounds from Alfalfa with Supercritical Carbon Dioxide

**DOI:** 10.3390/molecules26102994

**Published:** 2021-05-18

**Authors:** Olga Wrona, Katarzyna Rafińska, Aneta Krakowska-Sieprawska, Bogusław Buszewski

**Affiliations:** 1 Łukasiewicz Research Network—New Chemical Syntheses Institute, Al. Tysiąclecia Państwa Polskiego 13A, 24-110 Puławy, Poland; olga.wrona@ins.pulawy.pl; 2 Department of Environmental Chemistry and Bioanalytics, Faculty of Chemistry, Nicolaus Copernicus University in Toruń, Gagarina 7, 87-100 Toruń, Poland; katraf@umk.pl; 3 Centre for Modern Interdisciplinary Technologies, Nicolaus Copernicus University in Toruń, Wileńska 4, 87-100 Toruń, Poland; aneta_krakowska@wp.pl

**Keywords:** supercritical fluid extraction (SFE), bioactive compounds, *Medicago sativa* L., response surface methodology (RSM), optimization

## Abstract

The aim of this research was to provide crucial and useful data about the selection of the optimization criteria of supercritical carbon dioxide extraction of alfalfa at a quarter-technical plant. The correlation between more general output, including total phenolics and flavonoids content, and a more specified composition of polar constituents was extensively studied. In all alfalfa extracts, polar bioactive constituents were analyzed by both spectrometric (general output) and chromatographic (detailed output) analyses. Eight specific phenolic acids and nine flavonoids were determined. The most dominant were salicylic acid (221.41 µg g^−1^), ferulic acid (119.73 µg g^−1^), quercetin (2.23 µg g^−1^), and apigenin (2.60 µg g^−1^). For all seventeen analyzed compounds, response surface methodology and analysis of variance were used to provide the optimal conditions of supercritical fluid extraction for each individual constituent. The obtained data have shown that eight of those compounds have a similar range of optimal process parameters, being significantly analogous for optimization based on total flavonoid content.

## 1. Introduction

Due to its interesting physicochemical properties, carbon dioxide has been widely used in a supercritical state (scCO_2_) for the extraction of plant materials. It is an inexpensive, inert, tasteless, and odorless gas; is easily available; and is environmentally friendly. It does not contaminate the final product because under room temperature and reduced pressure, it changes the state into gas form and simultaneously leaves the extraction environment. Due to these advantages, carbon dioxide has been commonly used as a solvent in supercritical fluid extraction (SFE).

SFE is a popular separation technique of bioactive compounds from plant material applied successfully in industry, e.g., for hop or hemp extraction [1,2]. In supercritical extraction, the solvent properties and the main extraction parameters, such as temperature (T, K), pressure (P, MPa), and solvent flow rate (F, kg h^−1^), have a crucial impact on the composition and features of the obtained product. It has been proven that at lower values of temperature (T) and pressure (P) (but above the CO_2_ critical point), non-polar bioactive compounds are effectively extracted. As it was proven in earlier work, under higher values of P and T, polar bioactive components can be sufficiently separated from plant materials due to the small quadrupole moments of CO_2_ molecules and the diffusion of the constituents from the plant matrix [1]. The composition of the final product can vary depending on the values of the process parameters, which have a direct impact on the further application of obtained extract [1,2,3,4,5].

Nowadays, the main goal of the extraction in a supercritical state is not only to obtain the highest amount of the product, but also to enrich the extract with desired groups of bioactive compounds such as, e.g., fatty acids, lipids, phenolic acids, or flavonoids by providing the proper conditions [3,6,7,8,9,10,11,12,13,14,15]. The desirable final extract composition can only be achieved by the proper optimization. For SFE optimization, mathematical and statistical methods can be implemented. Due to the high cost of SFE, especially at a scale greater than laboratory scale, it is important to reduce the number of experiments. Box–Behnken design with response surface methodology (RSM) and analysis of variance (ANOVA) have commonly been applied for that purpose [3,9,10,11,12,13,14,15,16,17,18,19]. Response surface methodology (RSM) that generates polynomial quadratic Equation (1) was used to obtain the optimal process conditions:

(1) $$y=\beta_{0}+\sum_{i=1}^{n}\beta_{ii}x_{i}^{2\;\;}+\sum_{i<j}^{n-1}\sum_{j=2}^{n}\beta_{ij}x_{i}x_{j}+e$$

where *y* is an output variable; $\beta_{0},\;\beta_{i},\beta_{ii,\;}\;\mathrm{and}\;\beta_{ij}$ are coefficients of equation regression; $x_{i}x_{j}$ represent input variables; e represents error and residuals.

**Figure 1:**
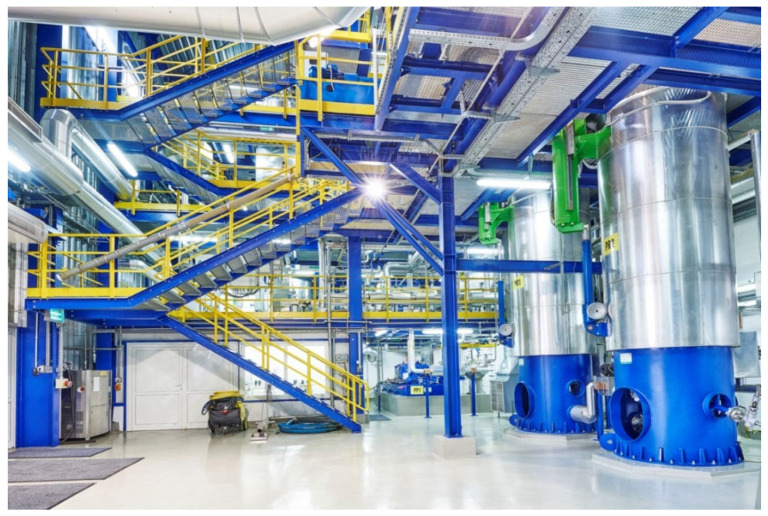
A modern industry plant of supercritical carbon dioxide extraction placed in Łukasiewicz Research Network—New Chemical Syntheses Institute (Puławy, Poland).

Optimization is a crucial step in upscaling the process, especially in industry (Figure 1). The optimization procedure has to be conducted properly. Firstly, the output value criterion should be defined. It can be the highest yield of the process, the final product enriched in the desired group of bioactive compounds, or the feedstock purified by the extraction of the undesirable group of compounds, such as pesticides or waxes. Secondly, the range of the main process parameters that have an influence on the chosen criteria has to be defined, such as temperature in the range between 313.15 and 353.15 K or pressure in the range of 20–80 MPa.

In this study, the impact of two approaches for the optimization of similar outputs determined by simple spectrophotometric and advanced chromatographic analyses was studied. An optimization procedure based on general criteria of supercritical fluid extraction with pure carbon dioxide at a quarter-technical plant was carried out previously and extensively described in our previous work [1]. *Medicago sativa* L. (alfalfa) was used as a feedstock due to its extensive spreading in Poland and accessibility in a proper amount for extraction at a scale larger than laboratory [1]. Moreover, this plant is very nutrient dense and has been considered a functional food. Lucerne is rich in bioactive compounds such as phenols, saponins, essential amino acids, or vitamins. Due to the versatility in the composition of bioactive compounds, it was possible to choose different optimization criteria [5,20,21,22,23]. In the previous paper [1], extractions were optimized to provide the highest yield, i.e., the maximum total contents of phenolics and flavonoids, where fast and inexpensive spectroscopic analyses were used for the determination of phenolics and flavonoids content. These optimization procedures ended successfully, providing the proper optimal parameters for obtaining maximum values of the chosen outputs [1].

In this paper, the main aim was to continue the study on the influence of chosen process parameters applied for SFE optimization on the more detailed and specified composition of the obtained extracts. Therefore, in this study, specific polar bioactive compounds were analyzed in all extracts obtained from lucerne using high-performance liquid chromatography coupled with mass spectrometry (HPLC-MS/MS) [22]. Moreover, for proper sample preparation for chromatographic analysis, a new method has been developed for the fractionation of polar and non-polar extracts’ components. HPLC is widely implemented in the determination of polar bioactive compounds from different matrices. Tocopherols, retinol, ester derivatives, and coenzyme Q10 were analyzed using high-performance liquid chromatography in milk and human plasma [23,24]. Flavonoids and phenolic acids were also determined using HPLC in orange juice and extract obtained from *Moringa oleifera* L. [25,26].

Response surface methodology and analysis of variance were implemented to obtain qualitative HPLC-MS/MS data of individual constituents to provide optimal conditions that result in the highest concentration of those compounds in the extract. RSM and ANOVA provide information about the proper fitting between the output (concentration of the individual components) and input variables (process parameters) and the relationship between the obtained model and the response variables. Moreover, the samples obtained under optimal conditions for the highest concentrations of TPC and TFC were also analyzed by chromatography due to the detailed composition of polar constituents. The obtained data allowed for correlating the results from spectrophotometric and chromatographic determinations and gave directions on how to efficiently carry out the optimization process at the industrial level.

## 2. Results

**Figure 2:**
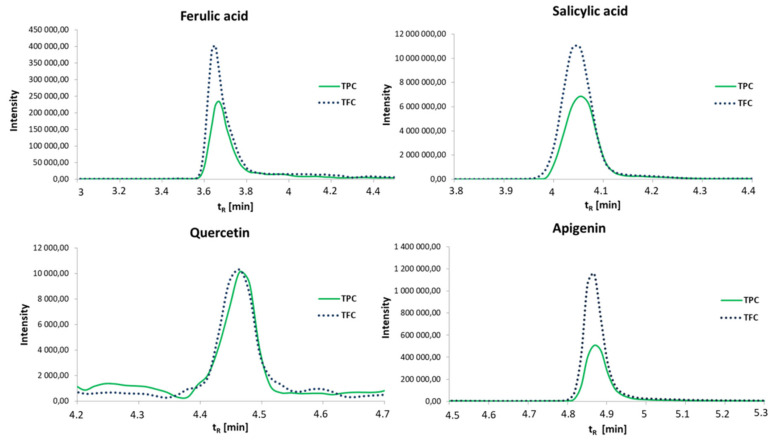
HPLC-MS/MS analysis results of designated polar constituents in alfalfa extracts.

All extracts obtained from lucerne at the quarter-technical plant in supercritical fluid extraction with carbon dioxide as a solvent were analyzed using the HPLC-MS/MS technique to simultaneously identify specific phenolic acids and flavonoids. The summarized results are listed in Table 1 and Table S1 (provided in the Supplementary Materials). An exemplary HPLC-MS/MS chromatogram for the most dominant compounds is shown in Figure 2.

Moreover, extracts obtained under the optimal conditions providing maximum values of total phenolics and total flavonoids contents as the main result of our previous work [1] were also analyzed for the determination of the same polar bioactive compounds from those two specific groups. Data with the detailed peak information are listed in Table 2.

**Figure 3:**
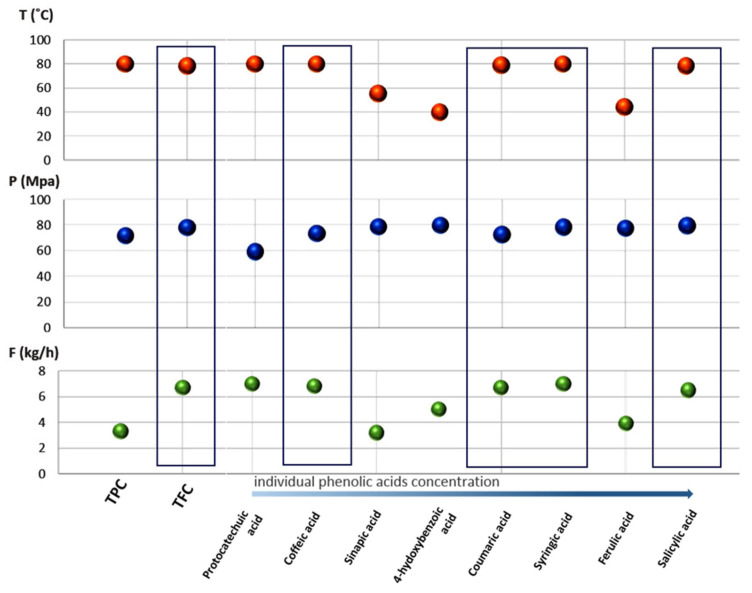
Comparison of optimal conditions of temperature, pressure, and scCO_2_ flow rate for TPC, TFC, and individual phenolic acids. The phenolic acids are ordered in increasing concentrations.

**Figure 4:**
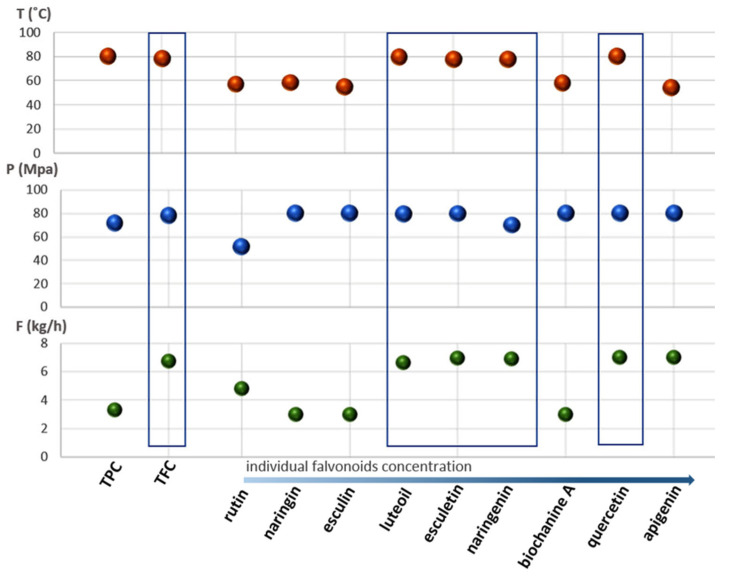
Comparison of optimal conditions of temperature, pressure, and scCO_2_ flow rate for TPC, TFC, and individual flavonoids. The flavonoids are ordered in order of increasing concentrations.

For all individual phenolic acids and flavonoids, response surface methodology and analysis of variance were applied (Supplementary Materials Table S2). The aim of the optimization was to obtain optimal conditions that provide the highest amounts of specific compounds in the final extract. The predicted values of individual polar components that should be obtained with extraction conducted using those optimized process parameters are listed in Table 3 and presented as Figure 3 and Figure 4, where squares indicate compounds with similar optimal ranges of process parameters providing the highest response. Response surface plots for the most dominant compounds are presented in Supplementary Materials in Table S2.

## 3. Discussion

In the HPLC-MS/MS analysis of samples from purified lucerne extracts, eight phenolic acids (coumaric, salicylic, caffeic, syringic, ferulic, protocatechuic, sinapic, and 4-hydroxybenzoic acids) and nine flavonoids (biochanin A, esculetin, esculin, naringenin, naringin, quercetin, rutin, luteolin, and apigenin) were determined (Table 1, Figure 2). From the phenolic acids group, the highest amounts of salicylic and ferulic acids were determined in all samples. Ferulic acid is a bioactive constituent of many foods that provide beneficial effects against disorders related to oxidative stress, e.g., cancer, diabetes, and neurodegenerative diseases [27,28]. Salicylic acid, as a phenolic compound, occurs in various plants where it has a significant role in protection against pathogenic agents. Moreover, the most common and well-described effect of salicylic acid is prostaglandin synthesis inhibition [29]. Apigenin and quercetin, as representatives of flavonoids, were the most dominant compounds in all alfalfa products. Apigenin and quercetin are natural bioactive flavone-type molecules that play an important role in the prevention and treatment of emerging global health issues such as diabetes or cancer. These effects are due to the physiological activity of those two flavonoids in the reduction in oxidative stress, inhibiting low-density lipoprotein oxidation and platelet aggregation, and acting as vasodilators in blood vessels [30,31].

Furthermore, based on the obtained data, a quite significant amount of apigenin was separated from lucerne by non-polar carbon dioxide in a supercritical state. The amounts of isolated compounds, not only apigenin but also quercetin, ferulic, and salicylic acids, are strongly correlated with the applied process parameters, especially high values of pressure, which determined the proper solubility and diffusion of a polar constituent from the plant material matrix [1,3,4].

Generally, based on the individual phenolics and flavonoids concentrations for each extract, in the product obtained by extraction under 333.15 K, 80 MPa, and 7 kg h^−1^, named E1, the highest amount of both apigenin and quercetin was determined: 2.60 and 2.04 µg g^−1^, respectively. Sample E2 was rich in salicylic acid, where the extraction was conducted under 353.15 K, 80 MPa, and 5 kg h^−1^. The process conducted under 333.15 K, 80 MPa, and 3 kg/h provided the product with the highest concentration of ferulic acid: 119.59 µg g^−1^. Depending on the type of compound, different values of the parameters of the extraction process determined the concentration of the isolated compounds (Figure 3 and Figure 4). Therefore, it was necessary to apply mathematical and statistical methods to compare the level of individual compounds with the total level of phenolic acids and flavonoids.

Response surface methodology (RSM) and analysis of variance (ANOVA) were used to study the chromatographic data for each individual compound. The main criterion was to obtain the process parameters for each analyzed compound that provide the highest concentration in the extract. For that purpose, Design Expert 11 was used. As a final result, optimal extraction conditions providing the highest value of response were obtained (Figure 3 and Figure 4). Simultaneously, the predicted values of the response were also verified. This predicted value was quantified from the regression curve after substituting the previously obtained optimal process parameters variables—T, P, and F. Those data are summarized in Table 3. Due to the high cost of supercritical carbon dioxide extraction at a half-technical scale, it was not possible to carry out the extraction with the parameters optimized for each determined compound. Therefore, the results obtained from the optimization of individual compounds were compared with results from the optimization of total phenolics and flavonoids that was published previously [1]. For coumaric, salicylic, caffeic, and syringic acids, the optimal temperature, pressure, and solvent flow rate were in high values of the analyzed range: T > 350.15 K, P > 70 MPa, and F > 6.5 kg h^−1^. This particular range of optimal process parameters’ values was also achieved for four flavonoids: esculetin, naringenin, quercetin, and luteolin (Figure 3 and Figure 4). These eight compounds from the seventeen analyzed had similar optimal extraction parameters or were in the similar range of analyzed T, P, and F. Moreover, for protocatechuic acid and apigenin, two of the obtained optimal parameters (T and F, P and F, respectively) had high values. For the remaining compounds, one of the optimized parameters was in the high range, except rutin, where all optimal parameters were in the mild range: 330.30 K, 51.62 MPa, and 4.82 kg h^−1^.

The content of individual phenolic acids and flavonoids analyzed by means of HPLC-MS/MS was correlated with the spectrophotometric results presented earlier. In our previous work, the parameters of extractions with scCO_2_ were successfully optimized to provide the highest total phenolics content and total flavonoids content. The optimal parameters were as follows: TPC = 353.1 K, 71.74 MPa, and 3.32 kg h^−1^; and TFC = 351.51 K, 78.29 MPa, and 6.72 kg h^−1^. In the rapid, cheap, and simple spectrometric analysis, the results obtained within the confidence interval were 21.92 mg GAE g^−1^ and 15.32 mg RU g^−1^ DM. For further comparison, a specific HPLC-MS/MS analysis was also performed for samples obtained using the optimized parameters (Table 2, Figure 3 and Figure 4). These obtained data allowed for comparing two approaches in the determination of chosen optimization criteria: general output, such as total phenolics or flavonoids content, and a more detailed one, such as the content of specific phenolic acids or flavonoids. The results are especially important from the industry point of view. Firstly, the general optimal conditions obtained as an optimization result for providing the highest amount of total flavonoids content in lucerne extract were in the same range of T > 350.15 K, P > 70 MPa, and F > 6.5 kg h^−1^ as obtained for eight individual compounds (coumaric, salicylic, caffeic, and syringic acids and esculetin, quercetin, naringenin, and luteolin). Secondly, the HPLC-MS/MS analysis showed that the summarized amounts of all determined compounds in extracts optimized for TPC and TFC were significant (for TPC, 258.11 µg g^−1^, and TFC, 347.91 µg g^−1^). However, in the TFC extract, there was almost 100 µg g^−1^ more of the determined phenolic acids and flavonoids than in the product optimized for TPC. The obtained results clearly indicate that simple total flavonoids content determination by the spectrophotometric method is more suitable for the optimization of SFE from plant material than total phenolics content. This simple and rapid method with Al_2_Cl_3_ reagent provides a great response as an optimization criterion, resulting in a high concentration of the polar bioactive constituent in the product.

Moreover, based on individual components’ concentration in extracts, in the TFC sample, quite significant amounts of coumaric acid, salicylic acid, synaptic acid, ferulic acid, naringenin, quercetin, and apigenin were obtained. The obtained results are promising for further optimization of the process, due to the compatibility between more general and specific results. The difference in the amount of polar bioactive constituents between the extract optimized for TFC (347.91 µg g^−1^) and the total predicted values for each individual compound (394.79 µg g^−1^) is not substantial. It is clear that in a more general determination of the optimization criteria through TFC measurement, significant amounts of both phenolics and flavonoids were determined. Analysis of the results indicates that spectrophotometric determination of flavonoids is a much better criterion for the optimization of extraction than the Folin-Ciocalteu method is. This criterion is also better for obtaining high-quality extracts compared to analysis of individual compounds because it allows for a reduction in costs. However, further analysis on the optimization of plant material extraction should be carried out to finally verify this hypothesis.

In the literature, there is no similar investigation. Firstly, due to the physicochemical properties of carbon dioxide, this solvent is not considered for the extraction of polar constituents. Secondly, the limitations of the applied devices play a huge role in this study. Commonly, extractions have been conducted under a range of pressure below 550 bar where, during the extraction with scCO_2_, non-polar bioactive compounds have been extracted easily due to their excellent solubility in carbon dioxide. However, in our study, a range of pressure values up to 800 bar was investigated. Those P values allow for investigating the separation of polar bioactive constituents when diffusivity (not only solubility) plays an important role in the extraction of bio-compounds. The obtained lucerne extracts had the consistency of a paste, which requires individual preparation of samples for HPLC-MS/MS analysis. Therefore, a new method has been developed for extract fractionating by dissolving in methanol, lowering the temperature, and centrifuging at reduced temperature to remove lipids to obtain a fraction of the polar active compounds. After that process, methanol was evaporated and the obtained extracts were dissolved in propylene glycol, which is the most desirable solvent in the cosmetic industry. In our previous work, extracts were determined by supercritical fluid chromatography (SFC), but for the analysis, raw extracts were used [1]. The discrepancies in determinations between SFC and HPLC-MS/MS result from differences in the preparation of samples for the two analyses. Due to their consistency, raw extracts from *M. sativa* were not suitable for further industrial processing and for HPLC-MS/MS analysis. Therefore, we developed a method that allows us to remove lipid compounds. From the qualitative point of view of analysis, the most extensive data about compounds were obtained by the HPLC-MS/MS technique. Moreover, lipid compounds obtained during fractionation are also very interesting and we plan to study their composition by GC-MS.

## 4. Materials and Methods

### 4.1. Chemicals and Reagents

Pure (min. 99.9% *v*/*v*) carbon dioxide was produced in Grupa Azoty Zakłady Azotowe “Puławy” PLC. All other reagents and standards solutions were purchased from Sigma Aldrich (Steinheim, Germany) and were of analytical grade.

### 4.2. Plant Material

The *Medicago sativa* L. used in this study was harvested in 2017 in Zalesie, Poland. Alfalfa was dried and ground into a fine powder with particle sizes of 1–1.6 mm. The moisture content of the plant material was 12.1% (*w*/*w*).

### 4.3. Extraction and Optimization

The Box–Behnken design was used to verify the effects of three factors on the specific determined components from alfalfa extracts: temperature—T (313.15–353.15 K, 40–80 °C); pressure—P (range up to 20–80 MPa); and solvent flow rate—F (3–7 kg h^−1^). The complete design comprised fifteen experimental steps under the different conditions (Table 4). All the results and statistical analyses were accomplished using Design Expert 11.0 (Stat-Ease, Inc., Minneapolis, MN, USA).

Supercritical extraction with pure carbon dioxide as the solvent was performed using a quarter-technical plant (SITEC-Sieber Engineering AG, Maur, Switzerland) placed in the Łukasiewicz Research Network—New Chemical Syntheses Institute in Puławy, Poland. Extraction was carried in 2019 as part of the project start-up. In this procedure, 200 g of ground dried lucerne was loaded into the 1-liter extraction vessel. The specific extraction procedure was well developed in previous work [3]. In summary, the required extraction parameters were provided in the installation; then, the scCO_2_ was passed through to the extraction vessel loaded with the proper amount of feedstock. After the indicated time (extraction time was a constant value for each experiment, and it was 1 h), the system was switched to work on the bypass for washing off the extract deposits remaining in the pipelines. Finally, after achieving process parameters equal to the environmental ones, the post-extraction residue was removed from the extractor, and the extract from the separator was collected and analyzed. Firstly, the extract accumulated in the separator was carefully collected by a separate drain due to the high pressure in the separator, and secondly, the remaining residue was collected with a spatula without using any solvent and placed in a closed container from which air was removed by nitrogen insufflating. The extract was stored at −80 °C until use for further analysis.

### 4.4. HPLC-MS/MS Analysis

Small portions of extracts were weighed and diluted in 1 mL of methanol. Samples were well mixed and centrifuged for 30 min at low temperature (4 °C) to remove lipids and fatty acids. The obtained supernatant was filtered, evaporated to dryness, and finally weighted. That obtained mass was our reference for further calculation. Analysis of phenolic compounds was conducted using a Shimadzu LC-MS 8050 (Tokyo, Japan) chromatographic system equipped with a binary solvent delivery system (LC-30 AD), controller (CBM 20A), an autosampler (SIL-30A), and a column thermostat (CTO-20AC). The chromatographic runs were conducted using a Kinetex F5 column (100 × 2.1 mm, 2.6 µm, Phenomenex, Torrance, CA, USA) at a temperature of 25 ± 1 °C. The chromatographic conditions were as follows: flow rate of 0.4 mL min^−1^, volume of sample injection: 10 µL, mobile phase A (0.1% formic acid in water) and mobile phase B (acetonitrile). The gradient program was 0–7 min, 0–80% B at 7–8 min, 80–80% B at 8–10 min, and 80–0% B. For instrument control, collection, and analyzing data, Lab Solution 5.8 software was used. MS/MS analysis was performed on a triple quadrupole in the m/z of 100 to 1000 (Shimadzu Europa GmbH, Duisburg, Germany) equipped with an electrospray ionization (ESI) source in positive and negative ionization modes. Multiple reaction monitoring (MRM) was used for qualitative and quantitative analyses of phenolic compounds [22]. For HPLC analysis, working solutions in the range 0.05–10,000 ng/mL were obtained by diluting the stock solution (100 µg/mL) with methanol and stored at −20 °C. The limit of detection (LOD) and the limit of quantification (LOQ) were determined for each standard. The LOD and LOQ are signal-to-noise ratios, three (S/N = 3) and ten (S/N = 10) times the noise level, respectively. The LOD and LOQ for biochanin A, esculin, naringenin, naringin, and rutin amounted to 0.01 and 0.033 ng/mL. The LOD and LOQ values for salicylic acid, esculetin, and apigenin were 0.1 and 0.33 ng/mL. Values ten times higher than these (1 and 3.3 ng/mL) were obtained for protocatechuic acid, sinapic acid, and quercetin, while for ferulic acid, these values were one hundred times higher (10 and 33 ng/mL). The LOD and LOQ values for luteolin were 0.05 and 0.165 ng/mL, respectively, while for coumaric acid and caffeic acid, these values were ten times higher (0.5 and 1.65 ng/mL). The LOD and LOQ for syringic acid and 4-Hydroxybenzoic acid were 50 and 165 ng/mL, respectively.

### 4.5. Total Phenolics Content (TPC) and Total Flavonoids Content (TFC)

The methods for obtaining the total phenolics content (TPC) and total flavonoids content (TFC) have been described in a previous paper [1]. Briefly, for TPC, 100 µL of lucerne sample was added to 1.5 mL deionized water and 100 μL Folin–Ciocalteu reagent. After 8 min, 300 μL of 20% sodium carbonate was also added to the vial. After 30 min at 20 °C in the dark, absorbance spectra were recorded at 765 nm against a blank. Results were expressed as mg gallic acid equivalents GAE g^−1^ DM. For the TFC, 0.25 mL of sample was mixed with 0.5 mL of 2% AlCl_3_ (in 96% ethanol solution) and diluted to a specified volume (1 mL) with EtOH. After 40 min at ambient temperature, sample absorbance was measured against a blank at 420 nm. TFC was expressed as mg rutin equivalents.

## 5. Conclusions

The obtained data are of great importance in the extraction processes on an industrial scale. There is no investigation similar to this, especially for the optimization of the SFE of polar bioactive compounds at a quarter-technical plant. Furthermore, the HPLC-MS/MS results provided detailed data about the extraction of a wide range of polar bioactive constituents from lucerne and proved that pure carbon dioxide, despite the non-polar character of the solvent, in a supercritical state can be efficient in the separation of phenolic acids and flavonoids from plant materials, albeit at high values of temperature and pressure: T > 350.15 K, P > 70 MPa. This conclusion is especially valuable from the industry point of view. In an industrial plant, there is no possibility to use a co-solvent for the extraction of polar bioactive constituents because installations are not equipped with proper pumps for passing a co-solvent through the feedstock. Moreover, the amount of co-solvent would be enormous, taking into account the mass of the feedstock, measured in tons. Finally, it would be a problem to evaporate the huge volume of the co-solvent from the obtained extract. However, the developed method of fractionation requires much less solvent.

In conclusion, optimization based on general criteria determined by a non-expensive and rapid spectrophotometric technique simultaneously provides a significant amount of individual phenolic acids and flavonoids. The more suitable analytical method for both groups of compounds was total flavonoids determination as opposed to total phenolics content determination. This study indicates that there is no significant reason to increase the cost of the optimization, especially in industry, by using a more specific and advanced technique for the extract determination due to the price of the purchase and exploitation of the HPLC-MS/MS device and the cost of analysis itself. The cost of analysis mainly consists in the cost of the working time of the analyst, the time of analysis itself, and the price of the used solvents and standards, which is much higher for chromatographic than spectrometric analysis.

## Figures and Tables

**Table 1:** Specific results of HPLC-MS/MS analysis of fifteen lucerne extracts, where quantities are expressed as µg g^−1^ of purified extract.

Compounds	Number of Extraction Experiments														
	E1	E2	E3	E4	E5	E6	E7	E8	E9	E10	E11	E12	E13	E14	E15
Coumaric acid	5.98	3.93	0.64	5.22	2.69	2.41	2.38	2.70	2.63	1.18	4.25	2.49	1.85	0.45	4.07
Salicylic acid	103.18	225.70	54.80	87.86	64.48	84.84	66.38	2.00	96.87	43.07	58.49	91.34	119.56	45.01	221.41
Caffeic acid	0.78	0.97	0.42	0.36	0.58	0.47	0.60	0.61	0.38	0.27	0.15	0.26	0.60	0.25	1.24
Syringic acid	9.64	6.81	3.34	8.13	5.07	5.56	4.97	5.18	7.79	4.49	4.59	4.99	6.84	2.34	6.75
Ferulic acid	108.73	101.86	26.89	119.73	69.04	74.12	78.90	75.64	117.76	32.10	73.91	78.69	119.59	34.60	87.75
Protocatechuic acid	0.31	0.30	0.19	0.29	0.25	0.22	0.24	0.25	0.23	0.17	0.12	0.09	0.26	0.07	0.43
Sinapic acid	1.32	1.27	0.23	1.52	0.95	0.86	0.99	0.95	1.05	0.25	0.27	0.33	1.62	0.24	0.80
4-hydroxybenzoic acid	0.86	0.97	2.36	1.17	1.86	1.12	1.93	2.02	3.16	0.51	n.d.	n.d.	n.d.	0.24	0.36
Biochanin A	0.34	0.12	0.04	0.10	0.15	0.08	0.16	0.15	0.14	0.06	0.05	0.16	0.45	0.07	0.19
Esculetin	0.22	0.12	0.07	0.07	0.11	0.08	0.10	0.11	0.13	0.09	0.04	0.03	0.06	0.08	0.15
Esculin	0.01	n.d.	n.d.	n.d.	0.01	n.d.	0.01	0.01	0.01	n.d.	0.01	0.01	0.02	n.d.	n.d.
Naringenin	0.28	0.18	0.03	0.09	0.16	0.14	0.17	0.17	0.15	0.07	0.02	0.12	0.23	0.03	0.26
Naringin	0.01	0.01	n.d.	0.01	0.01	0.01	0.01	0.01	0.01	0.01	n.d.	n.d.	0.02	n.d.	0.01
Quercetin	2.04	0.86	0.28	0.96	0.55	0.73	0.56	0.59	0.73	0.32	0.66	0.68	2.23	0.40	1.36
Rutin	n.d.	n.d.	n.d.	0.01	0.02	0.00	0.02	0.02	n.d.	n.d.	n.d.	n.d.	0.01	n.d.	n.d.
Luteolin	0.14	0.17	n.d.	0.02	0.05	0.08	0.05	0.05	0.10	n.d.	n.d.	0.01	0.13	n.d.	0.11
Apigenin	2.60	1.22	0.08	0.02	0.46	0.98	0.55	0.49	1.57	0.47	n.d.	0.89	2.19	0.22	1.33
Σ=	236.44	344.49	89.37	225.56	146.44	171.7	158.02	90.95	232.71	83.06	142.56	180.09	255.66	84.00	326.22

**Table 2:** HPLC-MS/MS analysis of extracts obtained under optimal conditions for TPC and TFC, where t_R_—retention time.

Compounds	t_R_ (min)	MRM (m/z)	µg g^−1^	
			TPC	TFC
Coumaric acid	3.550	163–93	1.93	5.50
Salicylic acid	4.054	137–93	157.77	220.63
Caffeic acid	3.177	179–134	0.31	0.66
Syringic acid	3.243	197–95	7.87	8.64
Ferulic acid	3.682	193–133	83.73	106.22
Protocatechuic acid	2.582	153–108	0.13	0.17
Sinapic acid	3.653	223–121	0.97	0.84
4-hydroxybenzoic acid	2.940	137–65	n.d.	0.07
Biochanin A	5.672	283–211	2.40	0.84
Esculetin	3.183	177–89	n.d.	0.10
Esculin	2.592	339–177	n.d.	n.d.
Naringenin	4.844	271–119	0.15	0.21
Naringin	3.528	579–271	0.01	0.01
Quercetin	4.462	301–227	1.53	1.76
Rutin	3.506	609–300	n.d.	n.d.
Luteolin	4.543	285–133	0.04	0.12
Apigenin	4.864	269–117	1.26	2.14
			Σ = 258.11	Σ = 347.91

**Table 3:** Optimal conditions for individual determined polar bioactive compounds obtained as a result of the application of the response surface methodology and predicted values.

Compounds	Optimal Conditions				Predicted Value, µg g^−^^1^
	T, K	T, °C	P, MPa	F, kg h^−1^	
Coumaric acid	352.02	78.87	72.61	6.7	6.14
Salicylic acid	351.45	78.3	79.8	6.51	242.29
Caffeic acid	353.1	79.95	73.45	6.81	1.307
Syringic acid	353.15	80	78.46	6.98	9.98
Ferulic acid	317.26	44.11	77.69	3.94	124.37
Protocatechuic acid	353	79.85	59.2	6.98	0.44
Sinapic acid	328.74	55.59	78.72	3.2	1.65
4-hydroxybenzoic acid	313.15	40	80	5.011	2.94
Biochanin A	331	57.85	80	3	0.36
Esculetin	351	77.85	79.88	6.97	0.23
Esculin	328.22	55.07	80	3	0.02
Naringenin	350.7	77.55	70.1	6.93	0.28
Naringin	331.5	58.35	80	3	0.019
Quercetin	353.15	80	79.99	7	1.99
Rutin	330.3	57.15	51.62	4.82	0.014
Luteolin	352.49	79.34	79.3	6.61	0.19
Apigenin	327.6	54.45	80	7	2.57
					Σ = 394.79

**Table 4:** Box–Behnken design for supercritical carbon dioxide extraction of lucerne in both coded (−1, 0, 1) and uncoded forms.

	Box–Behnken Design		
	T, K	P, MPa	F, kg h^−^^1^
E1	333.15 (0)	80.00 (1)	7.00 (1)
E2	353.15 (1)	80.00 (1)	5.00 (0)
E3	353.15 (1)	20.00 (−1)	5.00 (0)
E4	313.15 (−1)	50.00 (0)	3.00 (−1)
E5	333.15 (0)	50.00 (0)	5.00 (0)
E6	353.15 (1)	50.00 (0)	3.00 (−1)
E7	333.15 (0)	50.00 (0)	5.00 (0)
E8	333.15 (0)	50.00 (0)	5.00 (0)
E9	313.15 (−1)	80.00 (1)	5.00 (0)
E10	333.15 (0)	20.00 (−1)	3.00 (−1)
E11	313.15 (−1)	20.00 (−1)	5.00 (0)
E12	313.15 (−1)	50.00 (0)	7.00 (1)
E13	333.15 (0)	80.00 (1)	3.00 (−1)
E14	333.15 (0)	20.00 (−1)	7.00 (1)
E15	353.15 (1)	50.00 (0)	7.00 (1)

## Data Availability

Not applicable.

## Supplementary Materials

The supplementary materials are available online.

## Sample Availability

Samples of the compounds used in this study are available upon request from the authors.

## Abbreviations

ANOVA—analysis of variance; BBD—Box–Behnken design; F (kg h^−1^)—solvent flow rate; GAE—gallic acid equivalent; HPLC-MS/MS—high performance liquid chromatography coupled with tandem mass spectrometry; n.d.—not detected; P (MPa)—pressure; RU—rutin equivalent; RSM—response surface methodology; scCO_2_—supercritical carbon dioxide; SFE—supercritical fluid extraction; SF—supercritical fluid; T (K)—temperature; TFC (mg RU g^−1^)—total flavonoids content; TPC (mg GAE g^−1^)—total phenolics content.
