# Supplementary material for: Comparative Studies of Selected Criteria Enabling Optimization of the Extraction of Polar Biologically Active Compounds from Alfalfa with Supercritical Carbon Dioxide
Source: Molecules. 2021 May 18;26(10):2994. doi: 10.3390/molecules26102994 (PMC8157860; doi:10.3390/molecules26102994)
Supplement: Supplementary file 1 [file molecules-26-02994-s001.zip › molecules-1214179-supplementary.pdf]

Table S1. Specific HPLC-MS/MS results for lucerne extracts, where quantities as express as μg $100g^{-1}$ DW (DW – dry weight of the feedstock; 200 g).

compounds	number of extraction experiments														
	**E1**	**E2**	**E3**	**E4**	**E5**	**E6**	**E7**	**E8**	**E9**	**E10**	**E11**	**E12**	**E13**	**E14**	**E15**
coumaric acid	2.99	1.97	0.32	2.61	1.35	1.21	1.19	1.35	1.32	0.59	2.13	1.25	0.93	0.23	2.04
salicylic acid	51.59	112.85	27.40	43.93	32.24	42.42	33.19	1.00	48.44	21.54	29.25	45.67	59.78	22.51	110.71
caffeic acid	0.39	0.49	0.21	0.18	0.29	0.24	0.30	0.31	0.19	0.14	0.08	0.13	0.30	0.13	0.62
syringic acid	4.82	3.41	1.67	4.07	2.54	2.78	2.49	2.59	3.90	2.25	2.30	2.50	3.42	1.17	3.38
ferulic acid	54.37	50.93	13.45	59.87	34.52	37.06	39.45	37.82	58.88	16.05	36.96	39.35	59.80	17.30	43.88
protocatechuic acid	0.16	0.15	0.10	0.15	0.13	0.11	0.12	0.13	0.12	0.09	0.06	0.05	0.13	0.04	0.22
sinapic acid	0.66	0.64	0.12	0.76	0.48	0.43	0.50	0.48	0.53	0.13	0.14	0.17	0.81	0.12	0.40
4-hydroxybenzoic acid	0.43	0.49	1.18	0.59	0.93	0.56	0.97	1.01	1.58	0.26	n.d.	n.d.	n.d.	0.12	0.18
biochanin A	0.17	0.06	0.02	0.05	0.08	0.04	0.08	0.08	0.07	0.03	0.03	0.08	0.23	0.04	0.10
esculetin	0.11	0.06	0.04	0.04	0.06	0.04	0.05	0.06	0.07	0.05	0.02	0.02	0.03	0.04	0.08
esculin	0.01	n.d.	n.d.	n.d.	0.01	n.d.	0.01	0.01	0.01	n.d.	0.01	0.01	0.01	n.d.	n.d.
naringenin	0.14	0.09	0.02	0.05	0.08	0.07	0.09	0.09	0.08	0.04	0.01	0.06	0.12	0.02	0.13
naringin	0.01	0.01	n.d.	0.01	0.01	0.01	0.01	0.01	0.01	0.01	n.d.	n.d.	0.01	n.d.	0.01
quercetin	1.02	0.43	0.14	0.48	0.28	0.37	0.28	0.30	0.37	0.16	0.33	0.34	1.12	0.20	0.68
rutin	n.d.	n.d.	n.d.	0.01	0.01	n.d.	0.01	0.01	n.d.	n.d.	n.d.	n.d.	0.01	n.d.	n.d.
luteolin	0.07	0.09	n.d.	0.01	0.03	0.04	0.03	0.03	0.05	n.d.	n.d.	0.01	0.07	n.d.	0.06
apigenin	1.30	0.61	0.04	0.01	0.23	0.49	0.28	0.25	0.79	0.24	n.d.	0.45	1.10	0.11	0.67

Table S2. Response surface plots for the most concentered polar bioactive compounds in lucerne extracts as a function of main process parameters.

<table>
<tr><th></th><th>Response surface plot as a function of: pressure (P) and temperature (T)</th><th>Response surface plot as a function of: solvent flow rate (F) and temperature (T)</th><th>Response surface plot as a function of: pressure (P) and solvent flow rate (F)</th></tr>
<tr><td>Ferulic acid, μg g⁻¹</td>
<td>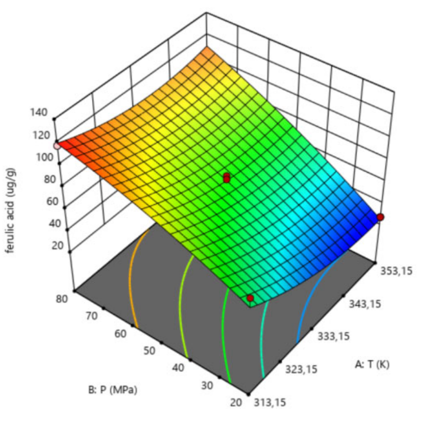
</td>
<td>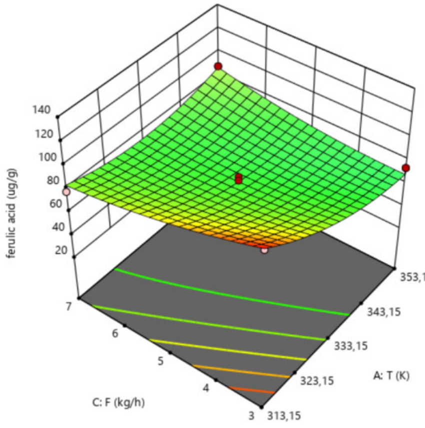
</td>
<td>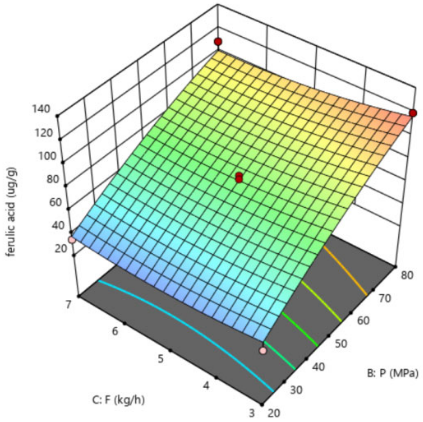
</td></tr>
</table>

Salicylic acid, $\mu g\ g^{-1}$

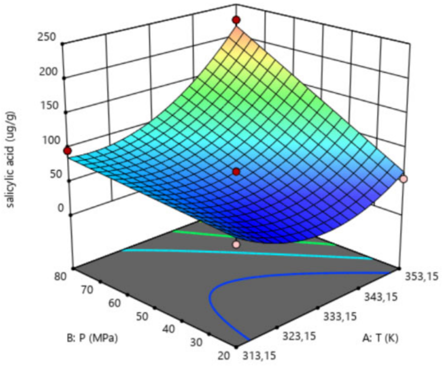

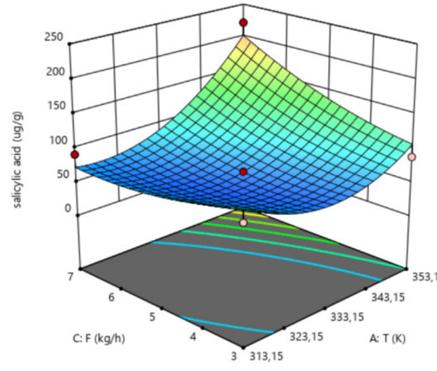

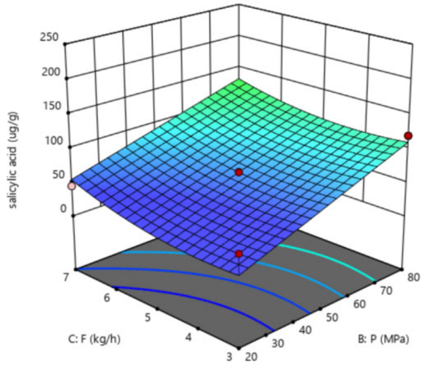

Quercetin, $\mu g\ g^{-1}$

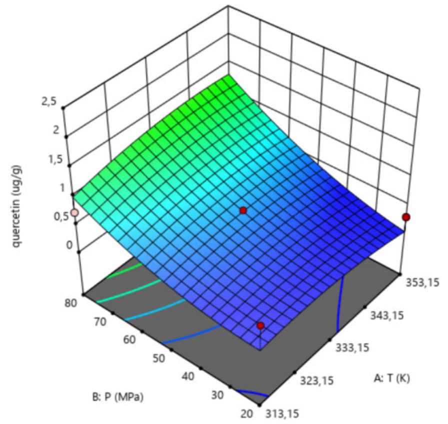

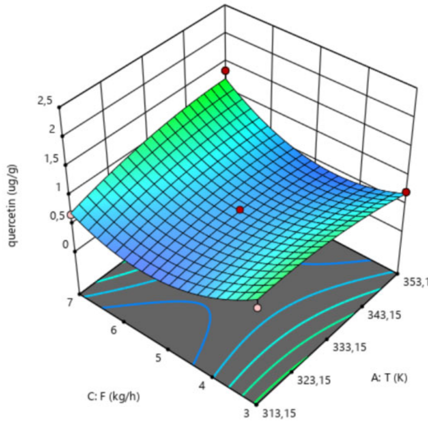

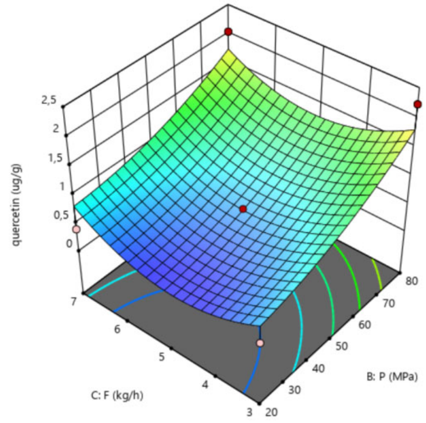

**Apigenin, µg g^{-1}**	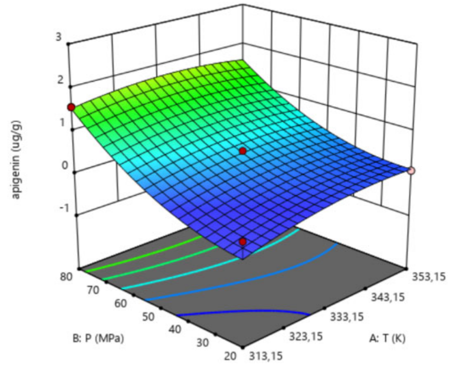	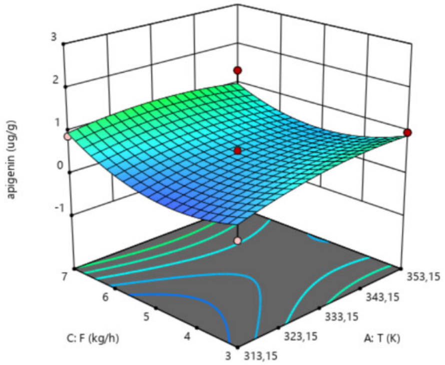	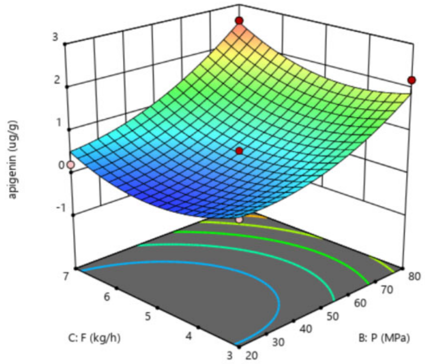
